# Supplementary material for: Genetic and Phenotypic Spectrum of KBG Syndrome: A Report of 13 New Chinese Cases and a Review of the Literature
Source: J Pers Med. 2022 Mar 5;12(3):407. doi: 10.3390/jpm12030407 (PMC8948816; doi:10.3390/jpm12030407)
Supplement: Supplementary file 1 [file Table S1 Craniofacial anomalies of 13 cases in this cohort.pdf › Table S1 Craniofacial anomalies of 13 cases in this cohort.pdf]

Table S1. Craniofacial anomalies of 13 cases in this cohort

	P1	P2	P3	P4	P5	P6	P7	P8	P9	P10	P11	P12	P13	
Craniofacial anomalies														12/12
Microcephaly	−	−	+	−	−	−	−	−	−	−	−	−	−	1/13
Triangular face	+	−	−	−	−	−	−	−	−	−	−	+	+	3/13
Abnormality of the hairline	−	−	−	−	+	−	+	+	−	−	−	−	−	3/13
Highly arched eyebrow/Broad eyebrow	+, broad eyebrow	−	−	−	+	−	+	+	+	+	−	−	+	7/13
Thick eyebrow/synophrys	−	−	−	−	+	+	+	+	+	−	−	−	−	6/13
Thick eyelashes	+	−	−	−	−	−	+	+	+	−	−	−	−	4/13
Hypertelorism	−	−	−	−	+	−	−	+	−	+	+	−	−	4/13
Epicanthus	−	−	−	−	+	−	−	+	+	+	+	−	−	5/13
Ptosis	+	+	+	+	−	−	+	−	−	−	−	−	−	5/13
Wide nasal bridge	−	+	−	−	−	−	+	+	−	−	−	−	+	4/13
Anteverted nares	−	+	−	−	−	+	+	+	−	−	−	−	+	5/13
Bulbous nose	−	−	−	−	−	−	−	+	−	−	−	−	+	2/13
Underdeveloped nasal alae	−	−	−	−	−	−	−	+		−	−	−	−	1/13
Short columella	−	−	−	−	+	−	−	−	−	−	−	−	−	1/13
Long/Smooth philtrum	+, long philtrum	−	−	−	−	−	−	+, Smooth philtrum	+	−	−	+	+	5/13
Thin upper lip	−	−	−	−	−	−	−	+	−	−	−	+	−	2/13
Cupid bow upper lip	−	−	−	−	−	−	−	+	−	−	−	−	+	2/13
Micrognathia	−	+	−	+	−	+	−	−	+	−	−	+	+	5/13
Preauricular skin tag	+	−	−	−	−	−	−	−	+	−	−	−	−	1/13
Preauricular pit	+	−	−	−	−	−	−	+	−	−	−	−	−	2/13
Abnormal location of the ear	−	−	−	+, Auricle supination	+, Asymmetry of the ears	−	−	−	−	+, Asymmetry of the ears	−	−	−	3/13

+, Positive Phenotype; −, Negative Phenotype; NA, Data non-available.
