# Supplementary material for: Genetic and Phenotypic Spectrum of KBG Syndrome: A Report of 13 New Chinese Cases and a Review of the Literature
Source: J Pers Med. 2022 Mar 5;12(3):407. doi: 10.3390/jpm12030407 (PMC8948816; doi:10.3390/jpm12030407)
Supplement: Supplementary file 1 [file jpm-12-00407-s001.zip › Table S3-Frequency of clinical phenotypes between 218 cases of truncated ANKRD11 variation and 13 cases of missense variation.pdf]

Table S3. Frequency of clinical phenotypes between 218 cases of truncated *ANKRD11* variation and 13 cases of missense variation

Clinical features	Truncated variation			Missense variation			*P* Value
	n	Positive	Negative	*n*	Positive	Negative	
**Craniofacial anomalies**	153	153	0	12	12	0	
**Dental anomalies**	159	150	9	9	8	1	0.432
Macrodontia of the upper central incisors	162	131	31	9	7	2	0.685
Other tooth anomalies	95	68	27	9	6	3	0.715
**Skeletal anomalies**							
Palatal irregularity	132	46	86	5	3	2	0.349
Limb anomalies	162	148	14	8	7	1	0.530
Spinal vertebral anomalies	98	48	50	8	5	3	0.716
Bone and joint of trunk anomalies	99	32	67	6	1	5	0.662
**Short stature**	195	96	99	13	10	3	0.083
**Growth retardation**							
(< 5 years old）Global developmental delay	149	138	11	12	7	5	0.003
(≥5 years old）Intellectual disability/learning difficulties	174	138	36	13	5	8	0.003
Behavioural anomalies	165	118	47	8	4	4	0.237
Delayed bone age	95	50	45	7	6	1	0.125
**Abnormality of the nervous system**							
Brain imaging anomalies	122	42	80	4	1	3	1
Epilepsy	105	45	60	6	0	6	0.079
EEG anomalies	42	14	28	5	1	4	1
**Visceral system anomalies**							
Congenital heart disease	162	44	118	6	3	3	0.351
Renal anomalies	26	12	14	2	0	2	0.492
**Gonadal anomalies**							
Cryptorchidism	63	28	35	6	2	4	0.690
**Ocular anomalies**	127	59	68	7	5	2	0.258
**Hearing loss**	167	60	107	8	4	4	0.466
