# Supplementary material for: Genetic and Phenotypic Spectrum of KBG Syndrome: A Report of 13 New Chinese Cases and a Review of the Literature
Source: J Pers Med. 2022 Mar 5;12(3):407. doi: 10.3390/jpm12030407 (PMC8948816; doi:10.3390/jpm12030407)
Supplement: Supplementary file 1 [file jpm-12-00407-s001.zip › Table S2-Clinacal and genetic analysis of 13 cases with missense variations.pdf]

Table S2. Clinical and genetic analysis of 13 cases with missense variations

Patient	P1	P2	P3	P4	P5	P6	P7 (P6′ mother)	P8	P9 (P8′ father)	P10	P11	P12	P13	
**Reference**	This study	This study	This study	Xu, M [14]	Walz, K. [5]	Miyatake, S. [28]		Murray, N. [29]		Kang, Y. [38]	Scarano, E. [40]	Guevara−Aguirre, J. [45]	Zhang, T. [50]	
**Gender**	Male	Female	Male	Male	Male	Male	Female	Female	Male	Male	Male	Female	Male	
**Age**	12y	3y6m	4y8m	20y	13y	7y	30y	6y	33y	14y	Na	5.7y	9y	
**Variant**	c.5519C>T (p.A1840V)	c.7832A＞T (p.H2611L)	c.6122T＞G(p.V2041G)	c.362T>A (p.M121K)	c.7535G>A (p.R2512Q)	c.6416C>T (p.P2139L)	c.6416C>T (p.P2139L)	c.3442G>A (p.G1148S)	c.3442G>A (p.G1148S)	c.2579C>T (p.S860L)	c.7534C>T (p.R2512W)	c.5786G>A (p.S1929N)	c.6427C>G (p.L2143V)	
**Variation source**	Mother	De novo	Father	De novo	De novo	Mother	NA	Father	NA	Mother	De novo	NA	Mother	
**GnomAD MAF**	0.00002	0	0	0	0	0.00005	0.00005	0.00003	0.00003	0.00004	0	0.000004	0.000004	
**Function predction**														
Provean	N(−0.938)	D(−8.491)	N(−1.307)	D(−4.715)	D(−3.298)	D(−3.769)	D(−3.769)	N(−0.487)	N(−0.487)	N(−1.540)	D(−6.596)	N(−0.590)	N（−0.473）	
PolyPhen2（HumDiv）	B(0.001)	PD (0.999)	B(0.007)	PD(0.892)	PD(1.000)	PD(1.000)	PD(1.000)	B(0.001)	B(0.001)	PD(0.996)	PD(1.000)	B(0.002)	PD	
MutationTaster	P	DC	P	DC	DC	DC	DC	P	P	DC	DC	P	P	
**Craniofacial anomalies**	+	+	+	+	+	+	+	+	+	Na	+	+	+	12/12
**Dental anomalies**	+	+	+	+/−	+	+	+	Na	+	Na	+	Na	−	8/9
**Skeletal anomalies**														
Hand/foot anomalies	−	+	+	+	+	+	−	+	Na	Na	+	Na	Na	7/9
Palatal irregularity	−	+	Na	+	Na	Na	Na	−	Na	Na	Na	Na	Na	2/4
Other Skeletal anomalies	+	−	−	+	+	+	−	−	Na	−	+	Na	Na	5/10
**Short stature**	+	+	+	+	−	+	+	−	−	+	+	+	+	10/13
**Growth retardation**														
Global development delay	+	Na	+	+	+	−	−	+	−	+	−	−	+	7/12
Mental retadation/ learning difficulties	+Mild learning difficult	+IQ74	−	−, IQ88	+	−	−	+IQ74	+	−	+IQ<80	−	−	5/13

Behavioural anomalies	+	+	+	Na	Na	−	−	−	Na	Na	+	Na	Na	4/7
Delayed bone age	+	−	Na	+	Na	+	Na	+	Na	+	+	Na	Na	6/7
**Neurological abnormalities**														
Epilepsy/EEG anomalies	Na	Na	−	EEG(+)	−	−	−	Na	Na	Na	Na	Na	Na	1/5
Brain imaging anomalies	−	Na	−	Na	Na	Na	Na	Na	Na	−	+	Na	Na	1/4
**CHD**	−	−	+	+	Na	+	−	+	Na	Na	−	Na	Na	4/8
**Ocular anomalies**	−	−	+	+	Na	+	+	+	−	Na	Na	Na	Na	5/8
**Cryptorchidism**	+	/	−	−	−	+	/	/	Na	Na	−	/	Na	2/6
**Hearing loss**	−	Na	+	+	Na	+	−	+	−	Na	−	Na	Na	4/8
**Kidney anomalies**	Na	Na	−	Na	Na	Na	Na	Na	Na	Na	−	Na	Na	0/2
**1st degree relative with KBG**	−	−	Na	−	−	+	+	+	+	Na	−	Na	Na	4/9
**Additional features**				9q31.2−q33.1microdeletion, Delayed puberty		IUGR		Volvulus				IUGR, Hypertrichosis	precocious puberty	

+, Positive Phenotype; −, Negative Phenotype; NA, Data non-available; D: Deleterious; N: Neutral; B: Benign; PD: probably damaging; P: polymorphism; DC: disease causing; Na, Data non-available.
